# Origin of Luminescent Centers and Edge States in Low-Dimensional Lead Halide Perovskites: Controversies, Challenges and Instructive Approaches

**DOI:** 10.1007/s40820-019-0254-4

**Published:** 2019-03-25

**Authors:** Jiming Bao, Viktor G. Hadjiev

**Affiliations:** 1 Department of Electrical and Computer Engineering, University of Houston, Houston, TX 77204 USA; 2 Department of Chemistry, University of Houston, Houston, TX 77204 USA; 3 Materials Science and Engineering, University of Houston, Houston, TX 77204 USA; 4 Texas Center for Superconductivity, University of Houston, Houston, TX 77204 USA; 5 Department of Mechanical Engineering, University of Houston, Houston, TX 77204 USA

**Keywords:** Low-dimensional perovskites, Luminescent centers, Edge states, Cesium lead halides, Deep-level states, Ruddlesden–Popper perovskites

## Abstract

With only a few deep-level defect states having a high formation energy and dominance of shallow carrier non-trapping defects, the defect-tolerant electronic and optical properties of lead halide perovskites have made them appealing materials for high-efficiency, low-cost, solar cells and light-emitting devices. As such, recent observations of apparently deep-level and highly luminescent states in low-dimensional perovskites have attracted enormous attention as well as intensive debates. The observed green emission in 2D CsPb_2_Br_5_ and 0D Cs_4_PbBr_6_ poses an enigma over whether it is originated from intrinsic point defects or simply from highly luminescent CsPbBr_3_ nanocrystals embedded in the otherwise transparent wide band gap semiconductors. The nature of deep-level edge emission in 2D Ruddlesden–Popper perovskites is also not well understood. In this mini review, the experimental evidences that support the opposing interpretations are analyzed, and challenges and root causes for the controversy are discussed. Shortcomings in the current density functional theory approaches to modeling of properties and intrinsic point defects in lead halide perovskites are also noted. Selected experimental approaches are suggested to better correlate property with structure of a material and help resolve the controversies. Understanding and identification of the origin of luminescent centers will help design and engineer perovskites for wide device applications.

## Highlights

- Controversial luminescent centers and edge states in low-dimensional perovskites were summarized.
- Evaluated experimental evidences and discussed the root cause for challenges and controversies.
- New experimental techniques were suggested to resolve the controversies and identify the nature of luminescent centers.

## Graphical abstract

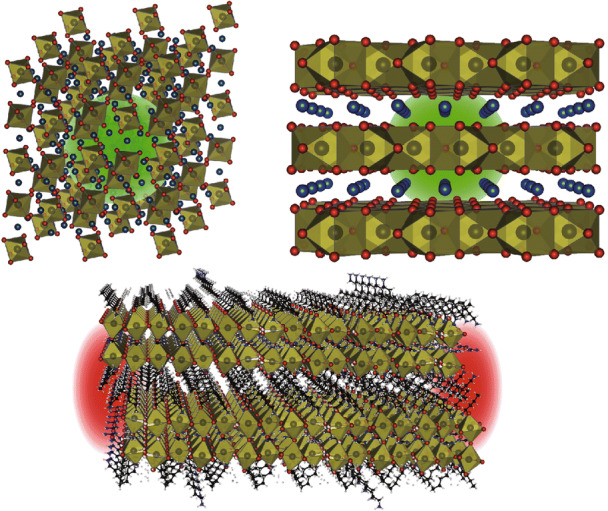

## Introduction

Lead halide perovskites have provided us not only a long-awaited material platform to realize the dream of high-efficiency solar cells and many other optoelectronic devices, but also a wide range of structures to explore unusual fundamental sciences [1–5]. Depending on spatial configurations, lead halide octahedrons can form structures from three-dimensional (3D) all the way to 0D perovskites [6]. While still not completely understood, the superior optoelectronic properties of perovskites are believed to originate from their immunity to defects and lack of non-radiative deep-level traps, which have made them ideal materials for high-efficiency low-cost solar cells and many other optoelectronic devices [1–5, 7]. This is another reason why recent observations of possible new types of luminescent states in low-dimensional perovskites are surprising; in particular, they have been used for higher efficient solar cells and even brighter light-emitting diodes [6, 8–18]. Such apparent deep-level luminescent centers have been observed in 2D CsPb_2_Br_5_ [19–24] and 0D Cs_4_PbBr_6_ [11, 15, 16, 25, 26]. Because of their optical property similarities to those of CsPbBr_3_ [27–29], the emission is believed to originate from embedded CsPbBr_3_ nanocrystals [15, 16, 30–36]. But many other researchers attribute it to intrinsic point defects because no CsPbBr_3_ nanocrystals have been found in their emissive samples [11, 25, 26]. The debates on the origin of Cs_4_PbBr_6_ are especially hot, as seen from four recent articles which acknowledge the controversy but are inclined to support one over the other [15, 16, 25, 26]. Similar deep-level luminescence centers have also been observed in low-dimensional organic–inorganic metal halide materials with better pronounced morphological dimensionality [37, 38] than that in Cs–Pb–Br system. The study of luminescence centers in structurally simpler all-inorganic lead halide perovskites, however, is expected to be instructive for further understanding the origin of these centers in all types of metal halide perovskites. As perovskites bring us more interesting properties and have found wide device applications, it is essential to understand the nature and mechanism for these luminescent centers.

In this mini review, the experimental evidences that support the opposing interpretations of the luminescence centers in Cs–Pb–Br system are analyzed, and challenges and root causes for the controversy are discussed. Selected experimental approaches are suggested to better correlate property with structure of a material and help resolve the controversies.

## Defects and Inclusions in Cs–Pb–Br System Rooted in the Ternary Phase Diagram

The perovskite-like compounds in Cs–Pb–Br system, CsPbBr_3_, CsPb_2_Br_5_ and Cs_4_PbBr_6_, can be easily synthesized via solution process or melt-grown [11, 19, 34, 39–42]. The possibility of the different phase coexistence is well expected, and the compounds are stable within narrow chemical potential ranges as shown by recent density functional theory (DFT) simulations [26].

**Fig. 1:**
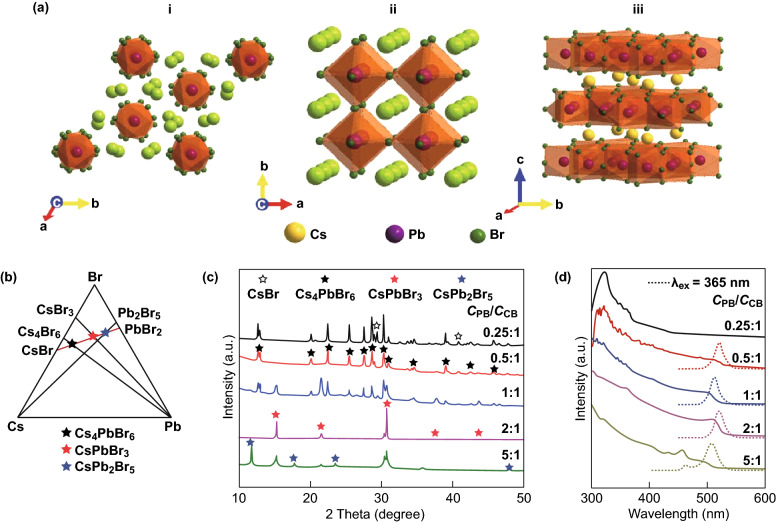
Influence of *C*_PB_:*C*_CB_ (*C*_PB_ = PbBr_2_, *C*_CB_ = CsBr = 0.04 M) on the composition and optical properties of Cs–Pb–Br nanocrystals. **a** Crystal structure of Cs_4_PbBr_6_ (i), CsPbBr_3_ (ii) and CsPb_2_Br_5_ (iii). **b** Ternary phase diagram of Cs, Pb and Br elements. Cs_4_PbBr_6_, CsPbBr_3_ and CsPb_2_Br_5_ fall on the line connecting PbBr_2_ and CsBr in the diagram. **c** XRD results at different *C*_PB_:*C*_CB_. **d** PL and UV–Vis absorption spectra of the nanocrystals prepared at different *C*_PB_:*C*_CB_. The excitation wavelength for PL spectra is 365 nm. Reprinted with permission from Ref. [43]. Copyright 2018 Royal Society of Chemistry

The ternary phase diagram of the Cs–Pb–Br system shown in Fig. 1b demonstrates that the three different perovskite-like structures can be produced using only CsBr and PbBr_2_ precursors. The different phases in Cs–Pb–Br system are grown by varying the precursor ratio (CsBr: PbBr_2_). As shown in Ref. [43], slight change of the crystal growth conditions and controlled precursor ratios can produce the low-dimensional phases CsPb_2_Br_5_ and Cs_4_PbBr_6_.

The 3D perovskite CsPbBr_3_ is the only compound in Cs–Pb–Br system that produces inherent green PL emission. This perovskite material was found unstable in moisture environment, and its instability has been used successfully to transform it into the lower dimension but stable phases CsPb_2_Br_5_ [20, 23] and Cs_4_PbBr_6_ [44] in water environment. The water-induced transformation of CsPbBr_3_ into CsPb_2_Br_5_ occurs in a sequential dissolution–recrystallization process under PbBr_2_-rich conditions [20]. Thus, synthesized CsPb_2_Br_5_ emits green photoluminescence (PL) with high PL quantum yield [20], but another approach using water was capable of growing non-emissive single crystals [23]. These observations hint that the green PL in CsPb_2_Br_5_ is likely due to highly luminescent CsPbBr_3_ nanocrystal remnants.

Zhang et al. [44] have grown successfully a millimeter-sized Cs_4_PbBr_6_ bulk single crystal in concentrated CsBr aqueous solution that lacks green luminescence emission [44]. In the same work, they also demonstrate that vacuum annealing treatment activates green PL in original nongreen-luminescent Cs_4_PbBr_6_ crystals, which was attributed to the possible formation of CsPbBr_3_. A reversible phase transformation between CsPbBr_3_ and CdPb_2_Br_5_ nanosheets under intense laser light has been demonstrated in Ref. [45]. Therefore, the narrow phase stability regions in the ternary phase diagram and the possibility of partially reversible phase transformations strongly support the expectations for foreign phase inclusions in the Cs–Pb–Br compounds.

### Luminescent State in 2D Wide Bandgap CsPb_2_Br_5_

**Fig. 2:**
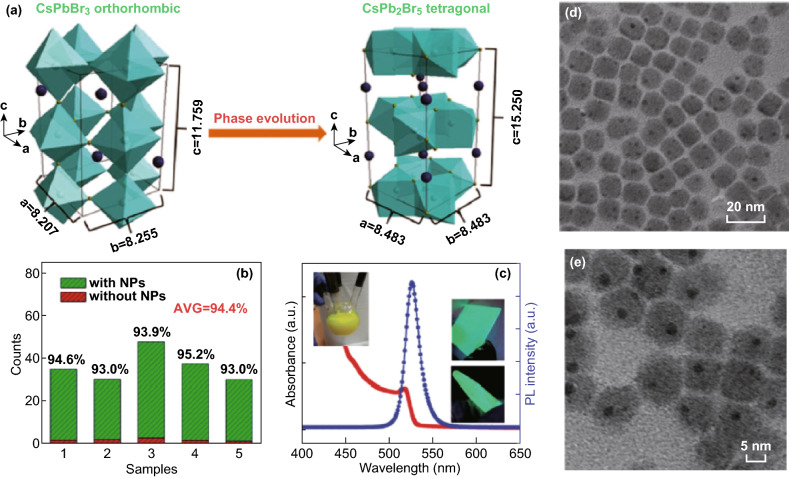
**a** Crystal structure schematics of the orthorhombic CsPbBr_3_ and tetragonal CsPb_2_Br_5_ (blue ball: Cs^+^). The unit of all the cell parameters is Å. Reprinted with permission from Ref. [22]. Copyright 2016 Royal Society of Chemistry. **b** The percentage of the CsPbBr_3_ nanocrystal being covered by CsPb_2_Br_5_ nanoparticles and their PL quantum yield (PLQY). **c** Absorption and PL spectra of CsPb_2_Br_5_/CsPbBr_3_ composites. The inset shows as-obtained products in four-necked flask and light emission of CsPb_2_Br_5_/CsPbBr_3_ deposited on the glass and tube excited with ultraviolet light. **d**–**e** TEM images of all-inorganic CsPb_2_Br_5_/CsPbBr_3_ nanocomposite at different magnifications. Dark dots in **e** are CsPb_2_Br_5_ nanoparticles. Reprinted with permission from Ref. [21]. Copyright 2016 Wiley–VCH

CsPb_2_Br_5_ is a layered lead halide structure with Pb-Br framework separated by Cs layers (Fig. 2a). 2D CsPb_2_Br_5_ has also attracted a lot of attention recently due to many conflicting reports on its luminescence although it was synthesized and studied long ago [41, 46]. Zhang et al. [21] were the first to report the beneficial effect of CsPb_2_Br_5_ to 3D all-inorganic perovskite CsPbBr_3_: the attachment of CsPb_2_Br_5_ nanoparticles to CsPbBr_3_ nanocrystals enhanced PL of CsPbBr_3_ by several folds and external quantum efficiency of CsPbBr_3_ light-emitting diodes (LEDs) by 50%. Figure 2b, c shows that more than 90% of CsPbBr_3_ are covered by CsPb_2_Br_5_ nanoparticles, but the PL and PLQY of CsPb_2_Br_5_/CsPbBr_3_ are nearly the same as those of pure CsPbBr_3_ nanocrystals. Figure 2d, e shows that these nanoparticles are not single phase, and high-resolution TEM reveals that they are CsPb_2_Br_5_/CsPbBr_3_ nanocomposites with dark smaller CsPb_2_Br_5_ nanocrystals attached to larger CsPbBr_3_ nanoparticles [21].

**Fig. 3:**
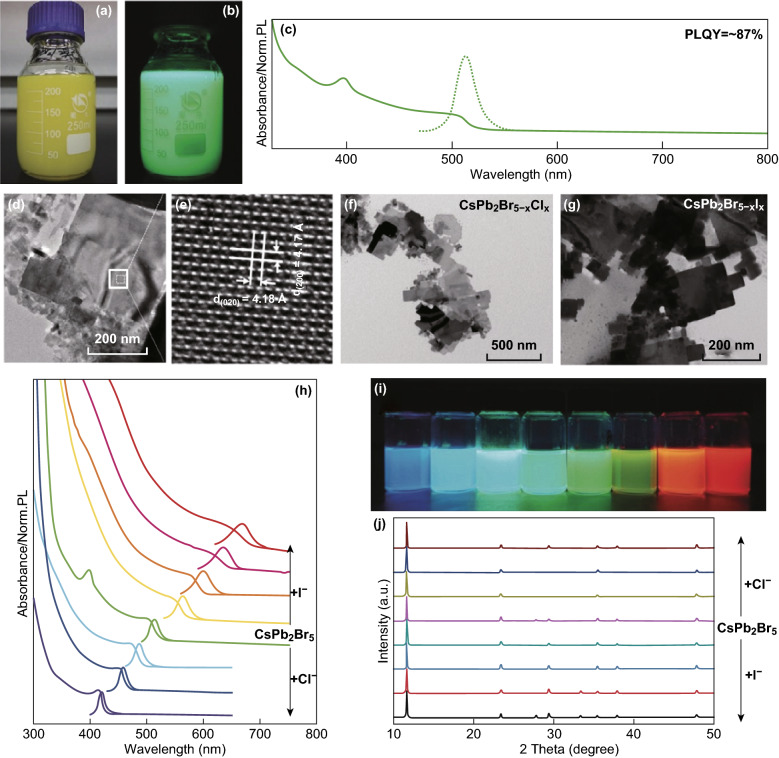
**a**, **b** Photographs of as-obtained colloidal CsPb_2_Br_5_ nanoplatelets suspension under ambient conditions and the UV light (365 nm) irradiation, respectively. **c** Absorption and PL spectrum of CsPb_2_Br_5_ nanoplatelets in toluene solution. **d**, **e** TEM and HRTEM images of CsPb_2_Br_5_ nanoplatelets, respectively. **f**, **g** TEM images of representative nanostructures of CsPb_2_Br_5−*x*_Cl_*x*_ and CsPb_2_Br_5−*x*_I_*x*_, respectively. **h** Evolution of the optical absorption and PL spectra of CsPb_2_Br_5_ nanoplatelets with increasing quantities of anion exchange of I^**−**^ and Cl^**−**^, respectively. **i**, **j** Corresponding photographs and powder XRD of the parent CsPb_2_Br_5_ nanoplatelet and anion-exchanged samples. Reprinted with permission from Ref. [19]. Copyright 2016 Wiley–VCH

Shortly after that, Wang and co-workers reported nearly 90% quantum efficiency of pure CsPb_2_Br_5_ nanoplatelets and subsequently expanded their emission wavelength to whole visible spectrum using ion exchange with I and Cl [19] (Fig. 3). Note that the purity of the initial CsPb_2_Br_5_ and ion-exchanged nanocrystals was verified by XRD and high-resolution TEM. Since then, many groups reported strong visible photoluminescence, high-efficiency LEDs, photodetectors and even lasing action in CsPb_2_Br_5_ microplates [20, 47–54]. Highly luminescent CsPb_2_Br_5_ nanowires with mixed halides are also synthesized recently [55].

**Fig. 4:**
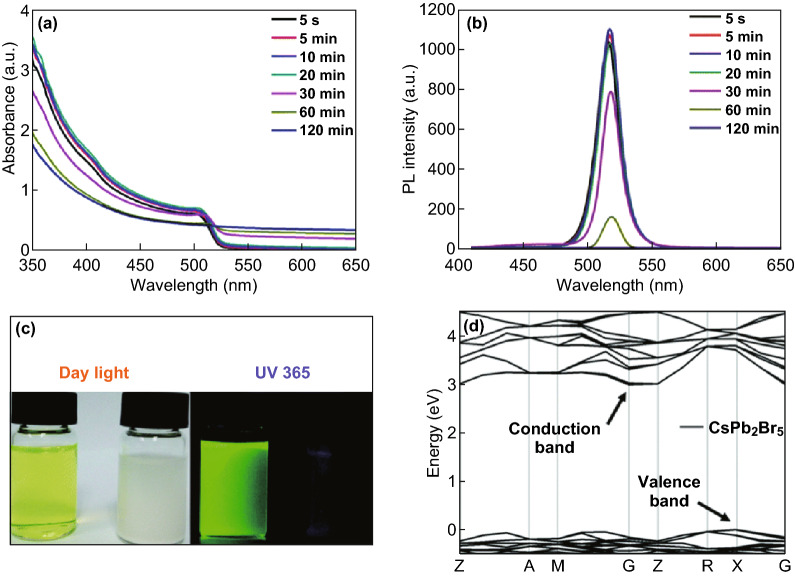
Synthesis of non-emissive CsPb_2_Br_5_ nanosheets from CsPbBr_3_ nanocubes. **a**, **b** Disappearance of absorbance and PL as the reaction time increases. **c** CsPbBr_3_ nanocube solution (after 5 s reaction time) and CsPb_2_Br_5_ nanosheet suspension (after 2 h reaction time) in toluene under daylight (left) and a UV lamp (365 nm, right). **d** Calculated electronic band structure of CsPb_2_Br_5_. Reprinted with permission from Ref. [22]. Copyright Royal Society of Chemistry

Despite numerous reports, the claim of highly luminescent CsPb_2_Br_5_ has been met with skeptics. Li et al. [22] synthesized CsPb_2_Br_5_ nanosheets from CsPbBr_3_ nanocubes. They have found that as the reaction goes on, both absorption and PL near 520 nm disappear, and the final product of CsPb_2_Br_5_ nanosheets displays no PL at all (Fig. 4). They also performed DFT simulation. The results (Fig. 4c, d) agree with the observation that CsPb_2_Br_5_ is an indirect wide band gap semiconductor [22].

**Fig. 5:**
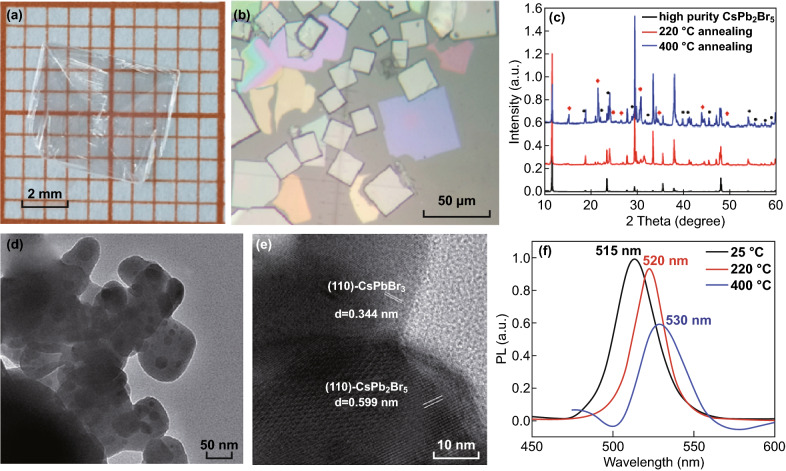
**a** A transparent colorless CsPb_2_Br_5_ crystal with an area of up to 5 × 5 mm^2^. **b** CsPb_2_Br_5_ flakes on sapphire. The thickness of the purple crystal is only 160 nm [13]. **c** Powder XRD pattern of CsPb_2_Br_5_ particles after annealing at different temperatures (red squares represent CsPbBr_3_ (PDF#18-0364), black dots represent PbBr_2_). **d** Low-resolution and **e** high-resolution TEM image of a representative CsPb_2_Br_5_ cluster after annealing at 400 °C. **f** PL spectra of the three CsPb_2_Br_5_ samples characterized by XRD in **c** under 400 nm excitation [14]. Reprinted with permission from Refs. [13, 14]. Copyright 2017–2018 Royal Society of Chemistry

The non-emissive nature of CsPb_2_Br_5_ can be best verified from transparent large-sized sheets in Fig. 5a, b [12, 13]. Emissive macro- or micro-CsPb_2_Br_5_ typically exhibits a characteristic yellow color as shown in Fig. 3a. Different colors of CsPb_2_Br_5_ sheets in Fig. 5b are due to their thickness-dependent optical interference under ambient or white light. As CsPb_2_Br_5_ can be produced by converting CsPbBr_3_ particles, CsPbBr_3_ particles can be recovered from CsPb_2_Br_5_ as well. Figure 5c shows the evolution of XRD patterns when high-purity CsPb_2_Br_5_ (black) was annealed at 220 °C (red) and 400 °C (blue). As the annealing temperature increases, X-ray pattern of CsPbBr_3_ particles begins to appear. This observation is also confirmed by TEM. Figure 5d, e shows CsPbBr_3_ particles attached on CsPb_2_Br_5_ in sample annealed at 400 °C. The change can also be seen in the PL spectra. The redshift of the PL band with annealing temperature is due to increasing size of CsPbBr_3_ particles [14]. Clearly, embedded CsPbBr_3_ particles in CsPb_2_Br_5_ can be a source for green PL emission in otherwise non-emissive pure CsPb_2_Br_5_. However, the same group has changed their mind and considered green emission as an intrinsic property of CsPb_2_Br_5_ after synthesizing and analyzing green emissive CsPb_2_Br_5_. Due to this reason, the mechanism for the green emission in CsPb_2_Br_5_ remains controversial. Many groups are aware of this controversy but are not able to support either of these two opposing claims [56–58].

### Luminescent State in 0D Wide Bandgap Cs_4_PbBr_6_ Perovskite

**Fig. 6:**
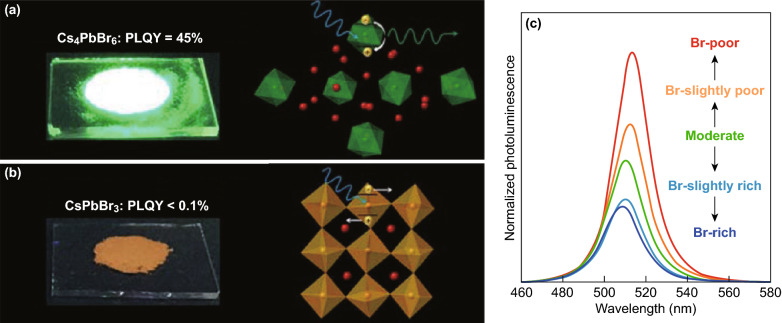
**a**, **b** CsPbBr_3_ and Cs_4_PbBr_6_ powders on the glass slides under UV light (365 nm). Insets are schematics of their crystal structures [11]. **c** PL intensity as a function of excitation wavelengths for Cs_4_PbBr_6_. (normalized PL spectra according to the absorbance at an excitation wavelength of 375 nm) [26]. Reprinted with permission from Refs. [11, 26]. Copyright American Chemical Society

In Cs_4_PbBr_6_, PbBr_6_ octahedrons are isolated by surrounding Cs ions and each octahedron behaves as a single molecular quantum dot (Fig. 6a inset), so Cs_4_PbBr_6_ is called 0D perovskite [15]. On the other hand, the crystallization of Cs_4_PbBr_6_ in a structure with translational symmetry and fixed orientation of PbBr_6_ octahedrons to each other clearly indicate that the spacing between PbBr_6_ is not enough to completely deactivate the interaction between them [16]. These interactions are better suppressed in the organic 0D metal halide hybrids [59, 60], which are closer to a quantum dot material. DFT calculations of an isolated Cs_4_PbBr_6_ structure, however, yield an energy gap close to those of bulk Cs_4_PbBr_6_ [61], which justifies the assignment of Cs_4_PbBr_6_ to 0D materials. Specifically, Cs_4_PbBr_6_ has attracted a lot of attention because of the high PL quantum efficiency reported in Ref. [11]. Figure 6 shows that Cs_4_PbBr_6_ also emits green light with a wavelength very close to that of CsPbBr_3_, but the PLQY is more than two orders of magnitude larger. Because Cs_4_PbBr_6_ is purified by dissolving CsPbBr_3_ contamination using dimethyl sulfoxide (DMSO), and no X-ray of CsPbBr_3_ is detected, the green emission is considered as an intrinsic property of Cs_4_PbBr_6_ [11]. Such strong green emission was initially attributed to the high exciton binding energy in isolated PbBr_6_ [11]. Later, an alternative explanation suggests that the green emission is due to a phonon-assisted transfer of photoexcited electrons to a charge-transfer state of Pb ions in the host lattice distorted by atomic displacements involved in the phonon [62]. Recently, the group published a series of papers and attributed the PL to intrinsic Br vacancies [26, 62–65]. Their theory has been supported by DFT calculations [26, 63] and other groups [25, 66–69].

**Fig. 7:**
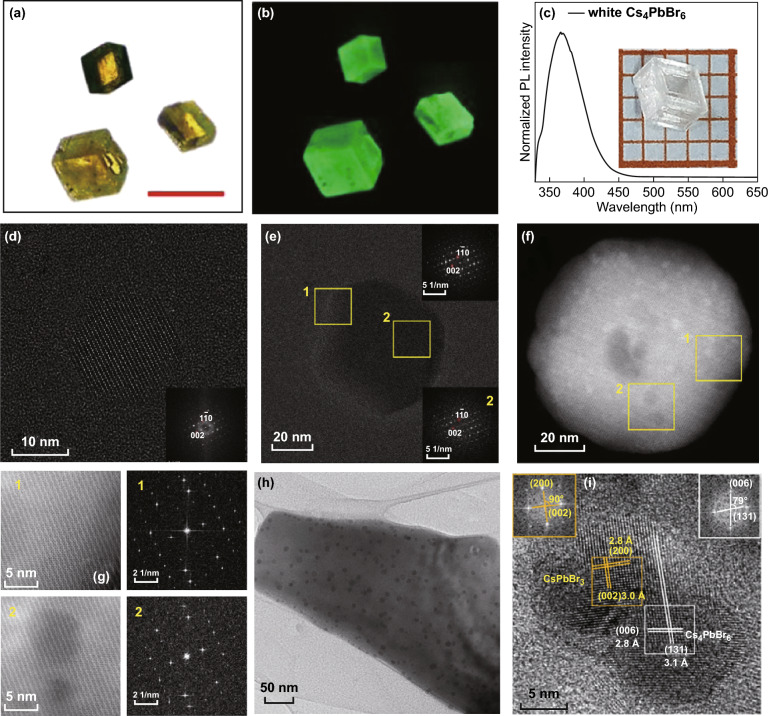
**a**, **b** Optical and fluorescent microscope pictures of emissive Cs_4_PbBr_6_ single crystals. Scale bar: 500 μm [63]. **c** Photograph of a millimeter-sized non-emissive Cs_4_PbBr_6_ single crystal. Inset: PL spectrum [44]. **d**–**g** Drift-corrected HRTEM images of **d** a non-emissive and **e** a green emissive Cs_4_PbBr_6_ nanocrystals. The FFT patterns are shown as insets. **f** High-angle annular dark field (HAADF)-STEM image of a green emissive Cs_4_PbBr_6_ nanocrystal and **g** HAADF-STEM images and FFT patterns of selected areas 1 and 2 in **f** [26]. **h** TEM and **i** HRTEM images of CsPbBr_3_ nanocrystals in the Cs_4_PbBr_6_ matrix. The insets show the FFT images [70]. Reprinted with permission from Refs. [26, 44, 63, 70]. Copyright American Chemical Society

The claim that the green PL emission is an intrinsic property of Cs_4_PbBr_6_ is also supported by the synthesis of large-sized single crystals (Fig. 7a, b). However, many other researchers do not agree with their observations and explanation [30–32, 44, 71]. Because the emission wavelength overlaps with that of CsPbBr_3_ very well, it has been believed that the strong PL originates from embedded CsPbBr_3_ nanocrystals. This alternative idea of non-intrinsic luminescent property is supported by the synthesis of non-emissive Cs_4_PbBr_6_, both large-sized single crystals (Fig. 7c) and nanocrystals [30, 44, 62, 71]. Opposing simulations also show that the Br vacancies cannot produce such deep-level defect states [5, 15, 16, 33]. As the strongest experimental evidences, both sides show high-resolution TEM images. Figure 7d–g compares TEM images of emissive and non-emissive Cs_4_PbBr_6_ nanocrystals. Both types of nanocrystals exhibit clean single crystal structure, and no CsPbBr_3_ inclusion is found. On the other hand, CsPbBr_3_/Cs_4_PbBr_6_ nanocomposites have been frequently synthesized and observed, and they exhibit strong PLQY as expected [16, 34, 70, 72].

### Bright Edge States in 2D Ruddlesden–Popper (R–P) Perovskites

Corner-sharing PbBr_6_ octahedrons as those in CsPbBr_3_ are definitely the structures that can produce visible PL. This has also been confirmed by the PL properties of two-dimensional (2D) R–P lead halide perovskites [10]. The observed crystal edge bright PL emission, different from that of the bulk one, in these materials is very instructive with demonstrated effects of PbBr_6_ framework relaxation at the surface of perovskite crystals.

A surface is an inevitable termination of periodic lattices of any single crystals even when we are only interested in their bulk properties. For 2D materials, edges will become surfaces and introduce surface defects as they terminate their 2D expansion. A surface will typically introduce detrimental or unwanted effects to the bulk materials so that surface treatment or passivation is crucial for the desired function or performance of materials. Because of this reason, it was very surprising that the edges of 2D organic–inorganic perovskites provide a deep-level luminescent center that also enhances the performance of solar cells [10].

**Fig. 8:**
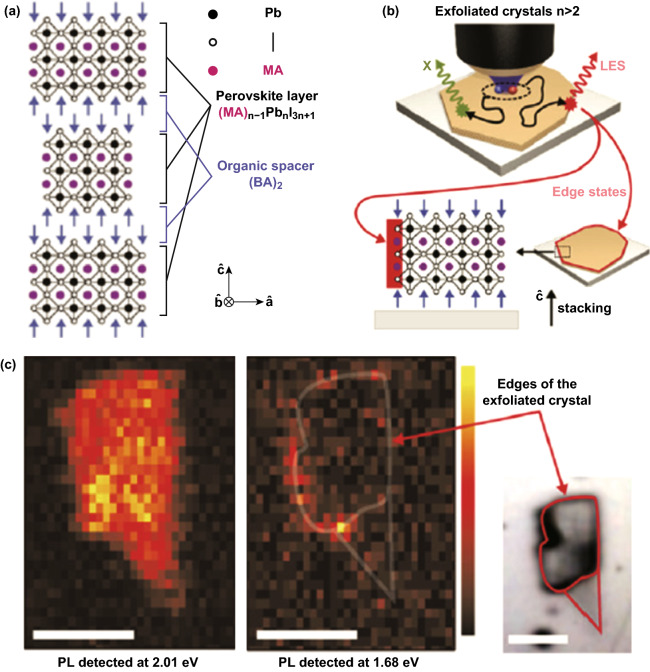
Edge emission in (BA)_2_(MA)_*n*−1_Pb_*n*_I_3*n*+1_ 2D R–P perovskite with *n* = 3. **a** Schematics of crystal structure. **b** Schematics of the photoabsorption and PL processes in 2D perovskite exfoliated crystals with *n* > 2. **c** Photoluminescence intensity map of a single exfoliated crystal. Scale bar is 10 μm. Reprinted with permission from Ref. [10]. Copyright American Association for the Advancement of Science

In 2017, Blancon et al. [10] reported that 2D R–P perovskites (BA)_2_(MA)_*n*−1_Pb_*n*_I_3*n*+1_ exhibit a low energy photoluminescence in the edge of exfoliated flakes when n is 3 or larger (Fig. 8a–c). Their emission energy is ~ 300 meV below the band gap of (BA)_2_(MA)_*n*−1_Pb_*n*_I_3*n*+1_. Unlike conventional deep-level defect states, they can quickly dissociate photoexcited excitons and prevent electron–hole from non-radiative recombination [10]. By fabricating 2D platelets vertically and having edges directly connected to the electrodes, the researchers have demonstrated 12% efficiency of 2D perovskite cells [10]. However, the nature of the edge states was not totally understood, and even their chemical composition and microscopic structure have not been experimentally identified in their initial report [10].

**Fig. 9:**
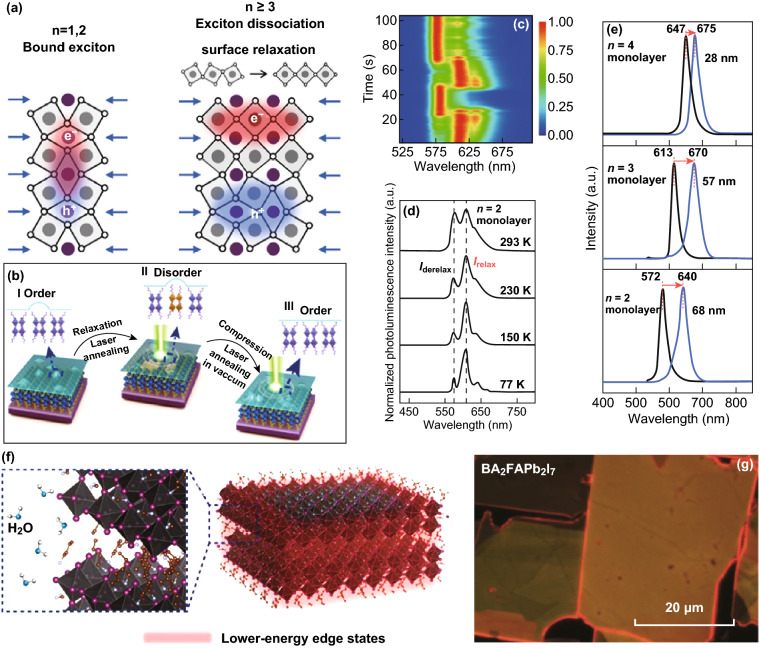
**a** Schematics of the surface-induced exciton dissociation in R–P perovskites with *n* ≥ 3. Reprinted with permission from Ref. [73]. Copyright American Chemical Society. **b** Schematic diagram showing the order–disorder transition by laser illumination. **c** Photoluminescence color map showing two continuous cycles of photoluminescence shifts during order–disorder transition, plotted as a function of laser irradiation time, emission wavelength and intensity. **d** Temperature-dependent changes in photoluminescence intensity. **e** Photoluminescence of *n *= 2–4 R–P perovskite monolayers. Black lines indicate the initial state photoluminescence, and blue lines are photoluminescence after relaxation. Reprinted with permission from Ref. [74]. Copyright Springer Nature. **f** Schematic of edge states of 2D halide perovskite due to moisture. **g** PL image of BA_2_FAPb_2_I_7_ excited by UV light. Reprinted with permission from Ref. [75]. Copyright American Chemical Society

It was not until a year later that a theory paper was published and offered a model to explain the edge states [73]. The calculation shows that when *n* > 2, the strain caused by the interface between inorganic and organic spacers will be relaxed to the edge lattices, causing a large lattice distortion (Fig. 9a). The distortion is large enough to create new localized state with energy much lower than the band gap [73]. However, there is still no experimental confirmation of the lattice distortion on the edge. A related paper was just published and reports the effect of organic spacer on the distortion of inorganic lattice in the surface of monolayer R–P perovskites [74]. Figure 9b–e demonstrates the sensitivity of electronic band structure to the Pb-I lattices and surface lattice relaxation. The edge emission was just confirmed in a very latest work [75]. But as shown in Fig. 9f–g [75], the edge emission is not due to the intrinsic strain; rather, it is induced by water molecules. Furthermore, the edge emission can also be observed when *n* = 2. Again, these are just experimental observations, and the underlying structure and mechanism are still not clear and require further research.

## Problems and Challenges in Revealing the Origin of Luminescence Centers in Lead Halide Perovskites

The current problems in identifying the origin of PL from in-gap luminescence centers are due to relatively large inconsistency in the results of both computational simulations and experimental characterization of lead halide perovskites. The basic properties of lead halide perovskites and expected intrinsic point defects have been studied extensively by using DFT. The first attempts of calculating the band structure of lead halide perovskites using local density functionals (LDA) and generalized gradient approximation (GGA) as GGA-PBE produced band gap values in accordance with the experimental ones. The top of valence band in Cs–Pb–Br 0D, 2D and 3D materials is composed of *p*-orbitals of Br with contribution from *s*-orbitals of Pb, whereas the bottom of conduction band is completely based on *p*-orbitals of Pb. Lead is a heavy metal known to possess strong spin–orbital coupling (SOC). The must-have inclusion of SOC in GGA-PBE calculations, however, results in a strong underestimate of band gap values in these materials. This also influences significantly the energy calculations of the native point defects (vacancies, interstitials and antisites) and defect complexes. The energy-level positions of different defects with respect to the band gap edges change with activating SOC. This makes quite dubious the assignment of certain defects as deep in-gap luminescence centers. The analysis of this problem has found its first solution in 2015 when Du [76] showed that the local density functionals used without SOC produce correct band gap values due to error cancelations [77], whereas inclusion of SOC involves self-interacting errors and requires the use of screened hybrid functionals as Heyd–Scuseria–Ernzerhof (HSE) to reproduce correctly both the band gap and the energy position of defects. Kang and Wang [5] presented the first complete calculations of the formation energy of all type point defects in CsPbBr_3_ using HSE + SOC. The formation energy of defects was calculated taking into account the Fermi energy and atomic chemical potentials of constituents [78, 79]. Although this work seems to deliver solid results, a few details are alarming and indicate that it may not be the final word on solving problems of modeling defects in lead halide perovskites.

The HSE functional includes a portion of non-local Hartree–Fock (HF) exchange in addition to local GGA-PBE one. The HSE functional partitions the Coulomb operator for a pair of charges into two ranges: short (SR) $= \left[ {1 - erf\left( {\omega r} \right)} \right]/r$ and long (LR) $= erf\left( {\omega r} \right)/r$ that are defined and controlled by the range-separation parameter *ω* set empirically to 0.15 Bohr^−1^ in the so-called HSE03 [80] version and to 0.11 Bohr^−1^ in the HSE06 version. HSE incorporates 25% SR HF exchange (mixing parameter, *a *= 0.25), no LR HF exchange, 75% SR and full LR PBE exchange, and 100% PBE correlation. Test calculations using HSE03 [80] with *a *= 0.25 and *ω *= 0.15 Bohr^−1^ have reproduced well the band gap of a large number of semiconductors [81], that is, HSE is believed to be a universal functional. The band gap of CsPbBr_3_, however, is calculated correctly using HSE + SOC only with HF exchange portion *a* set to 0.43 [5]. Recent extension of HSE + SOC to calculations of 2D CsPb_2_Br_3_ and 3D Cs_4_PbBr_6_ shows that there are no universal HSE06 parameters *a* and ω that produce the band gaps correctly for all Cs–Pb–Br compounds [26]. The band gap of CsPb_2_B_5_ and Cs_4_PbBr_6_ is calculated to be close to the experimental one for *a *= 0.2 [26], that is, different from both *a *= 0.43 for CsPbBr_3_ and most importantly different from *a *= 0.25 of the original HSE06 that has been claimed to be a universal for correct calculations of band gaps in semiconductors. One yet unexplored path is to repeat these calculations with fixed original *a *= 0.25 in HSE functional and varying the screen parameter ω and then find a physical reason for different screening parameters in Cs–Pb–Br compounds.

**Fig. 10:**
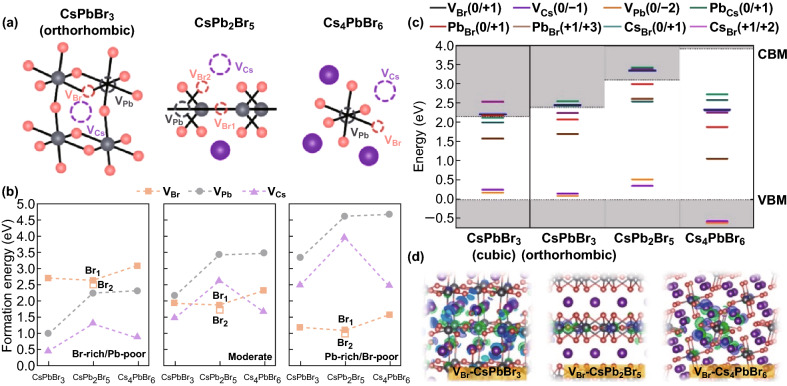
**a** Illustrations of Br, Pb and Cs vacancies (VBr, VPb and VCs). **b** Calculated defect formation energies for orthorhombic-CsPbBr_3_, CsPb_2_Br_5_ and Cs_4_PbBr_6_ at Br-rich/Pb-poor, moderate and Pb-rich/Br-poor conditions. **c** Defect charge transition levels of CsPbBr_3_, CsPb_2_Br_5_ and Cs_4_PbBr_6_. **d** Charge density distributions of *V*_Br_ defect states for CsPbBr_3_, CsPb_2_Br_5_ and Cs_4_PbBr_6_ calculated at the GGA/PBE level of theory. The band gaps were corrected using the HSE + SOC method. Reprinted with permission from Ref. [26]. Copyright American Chemical Society

The DFT results of defect formation energy calculations of CsPbBr_3_, CsPb_2_Br_5_ and Cs_4_PbBr_6_ and the defect energy levels with respect to the energy band gap in these compounds are shown in Fig. 10 [26]. The defect formation energy was calculated in a similar way as in Ref. [5], but the results for some of the defects in CsPbBr_3_ are different in the two papers. No critical analysis has been done so far on whether this is the most reliable and realistic approach for calculations of the defect formation energy. Apart from the supercell size-independent errors in these calculations as the choice of DFT functional and the choice of exchange correlation potentials discussed above, there are a number of supercell size-dependent errors, e.g., in Ref. [82], that have not been explored yet in the Cs–Pb–Br compounds. There is a need for a more rigorous approach to defect property calculations for the Cs–Pb–Br system in accordance with analyses and prescriptions given in Ref. [83].

The origin of green PL in Cs_4_PbBr_6_ is attributed to Br vacancies, *V*_Br_ (as in Ref. [26]). As shown in Fig. 10c, however, *V*_Br_ (0/+ 1) cannot be involved in the green PL emission observed in CsPb_2_Br_5_. The only candidates for defect mediated PL in CsPb_2_Br_5_ are the antisites Pb_Br_ and Cs_Br_. Another computational study of CsPb_2_Br_5_ [33], however, predicts the positions of unoccupied and occupied levels of *V*_Br_ (0) to lay at ~ 0.25 and ~ 0.5 eV, respectively, below the conduction band edge. The puzzle of the very similar green PL in CsPb_2_Br_5_ and Cs_4_PbBr_6_ due to defects remains unsolved. These notes show the complexity involved in the modeling of Cs–Pb–Br compounds and their native point defects and the degree of confidence one may have in the DFT results.

The arguments in favor of green PL in CsPb_2_Br_5_ and Cs_4_PbBr_6_ due to CsPbBr_3_ nanocrystal inclusions are better justified experimentally than those in support of native point defects. Indeed, the studies of CsPbBr_3_ absorption and emission spectra variation with nanocrystal size clearly show a quantum dot size effects with a PL peak position shift from 2.35 eV in bulk crystals to 2.7 eV in ~ 4-nm crystal [28, 84].

**Fig. 11:**
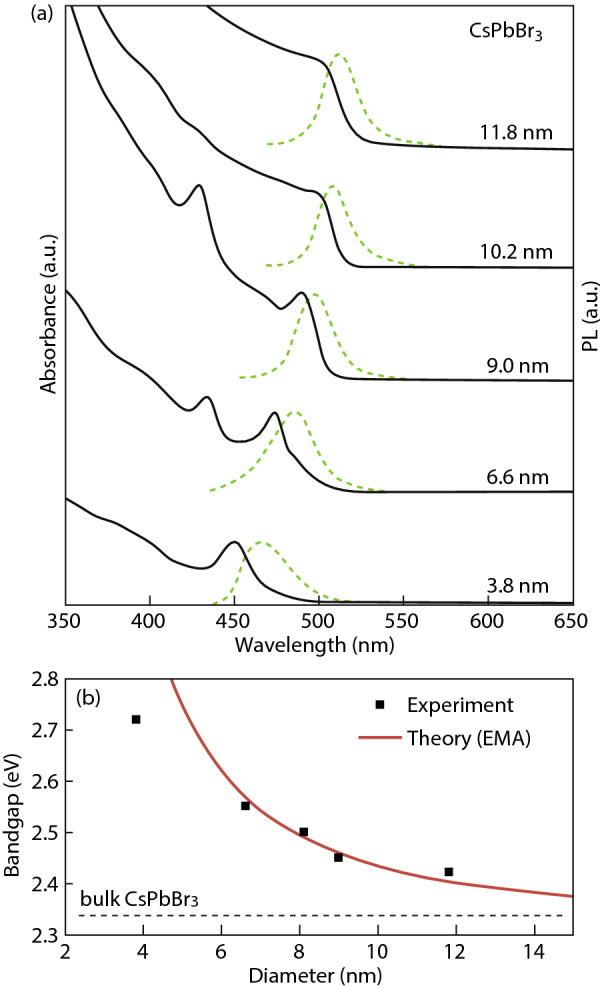
**a** Quantum size effects in the absorption and emission spectra of 5–12-nm CsPbBr_3_ NCs. **b** Experimental versus theoretical (effective mass approximation) size dependence of the band gap energy. Reprinted with permission from Ref. [28]. Copyright American Chemical Society

The results in Fig. 11 show that typically observed green PL luminescence at 2.35–2.5 eV in CsPb_2_Br_5_ and Cs_4_PbBr_6_ may well be due to CsPbBr_3_ nanocrystal inclusions in these wide band gap semiconductors. One way to move forward in revealing the nature of luminescence centers is to provide stronger experimental evidence on the nature of PL centers in Cs–Pb–Br system although this is also challenging as we discuss it below.

## Designing Combined Experimental Characterization Approaches for Better Consistency

Structure–property relation is the major goal of materials science and engineering. Specifically for the Cs–Pb–Br system one of the important properties is the photoluminescence, while its structure is determined by XRD, TEM and Raman spectroscopy. The last one is an indirect structure-related technique. The major reasons for these structure–property controversies or challenges are the difference in probing length scale and sensitivity. XRD is an average technique and has relatively low phase detection sensitivity. It is sensitive to the minority phase domain size, which below a few hundred nanometers usually diminishes and broadens the related XRD peaks, whereas PL can be emitted even from quantum dots and single molecular inclusions. TEM can provide detailed atomic structures, but there are two major difficulties. The first is that the size of PL and TEM probes are orders of magnitude different. TEM can only probe a much smaller sample, on the order of 100 nm in size; however, micro-PL still require a micrometer-sized sample. The second reason is that perovskite-like materials are very sensitive to electron beams and can get damaged easily, so the structure of perovskite is hard to be studied and advanced low-dose TEM is needed [65].

Raman is a well-established and sensitive technique to identify a material. In addition, Raman is compatible with PL and requires a small sample amount, so Raman can serve both the structure determination and property related to PL. For instance, the non-resonant Raman scattering is structure related, but at resonance it may probe electronic states that concurrently take part in PL. Raman and PL have been separately used in characterizing the luminescent centers, but they were only used for qualitative study, and a combined and calibrated Raman–PL has been missing. The key to such quantitative Raman–PL analysis is the calibrations of both Raman and PL using well-known reference materials. Note that PL is very sensitive to material quality, so a reference sample should be carefully chosen for the combined Raman and PL. For instance, CsPbBr_3_ nanocrystals can have a PLQY of 60–90% [27–29], but the PLQY of CsPbBr_3_ micropowders can be as low as 0.1%. CsPbBr_3_ nanocrystals should be used to confirm whether they are the source for green emission. So far, a successful application of combined Raman/PL mapping of the same sample area of CsPb_2_Br_5_ was reported in Ref. [23]. In that study, the Raman spectroscopy distinguishes the single crystalline part of CsPb_2_Br_5_ [77], which turned out to be non-emissive, from the polycrystalline part that produces green PL.

**Fig. 12:**
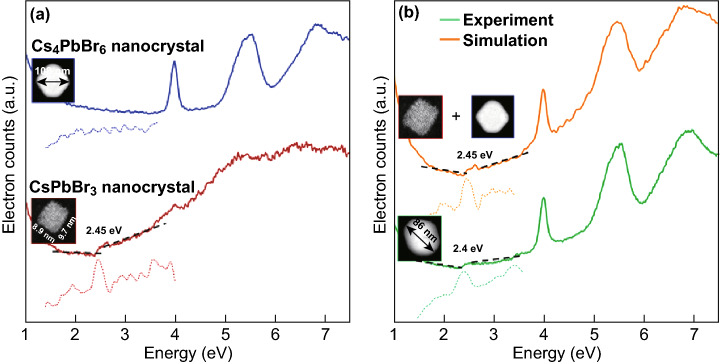
Energy band gap and structure. **a** Valence-loss EEL spectra representing the absorption of a CsPbBr_3_ nanocube (red spectrum) and a Cs_4_PbBr_6_ NC (blue), appearing in the same sample (160 °C). The band gap energy of the nanocube (2.45 eV) is determined from the peak of the first derivative of the EEL spectrum, which appears due to the abrupt onset in absorption (indicated by the dotted lines). No onset is observed for the Cs_4_PbBr_6_ NC which is an insulator with a large band gap energy of 4 eV. **b** Valence-loss EEL spectrum of a spherical nanocrystal (green) observed in the same sample, formed upon the hybridization of a nanocube and a nanohexagon. The latter process is represented by the simulated EEL spectrum (orange) which is obtained from adding the experimentally obtained spectra of the nanohexagon and nanocube as shown in **a**. The hybrid has, as expected, a band gap energy similar to that of the nanocube. Reprinted with permission from Ref. [31]. Copyright American Chemical Society

The optical absorption spectra of Cs–Pb–Br compounds can indicate indirectly, but not for certain, whether the samples are PL emissive or not. A promising alternative approach reported in Ref. [31, 85] uses TEM for structural characterization and energy loss spectroscopy (EELS) in the low-loss region as equivalent of optical absorption in CsPbBr_3_ and CsPbBr_3_/Cs_4_PbBr_6_. The results of TEM–EELS characterization of CsPbBr_3_/Cs_4_PbBr_6_ nanocrystals are shown in Fig. 12. The main limitations of the EELS technique are the effect of sample thickness, relatively low sensitivity and energy resolution in the low-loss region when compared to optical spectroscopy.

The ultimate technique for structure–property relation is combined TEM-PL on a single nanocrystal [86]. The Raman scattering from ultra-small amount of highly luminescence compounds could be too weak to be detectable. A major challenge is to avoid electron beam-induced damages to perovskites. High-resolution TEM imaging has been used by competing sides to support their arguments, but no such combined study was reported. The observation of CsPbBr_3_/Cs_4_PbBr_6_ nanocomposites certainly cannot exclude possible defect luminescent states; although no apparent CsPbBr_3_ nanocrystal was found in some single crystals of emissive Cs_4_PbBr_6_, PL from the same nanocrystal was actually not demonstrated [19, 51, 55]. More importantly, PL should be performed before and after TEM imaging to ensure no damage has occurred. To reveal the origin of edge states in R–P perovskites [10, 75], the next step is to reproduce the reported results and further determine the factors that are responsible for the edge states. Besides the reported TEM and AFM [75], noninvasive techniques such as Raman and FTIR should be used to identify the structural and chemical changes to the edge lattices [85, 87]. Note that nanometer scale versions of Raman and FTIR are already available to probe local structures [88, 89].

PL, Raman, XRD and TEM are passive techniques; new techniques that can apply external stimulus such as mechanical, electrical, or magnetic force to probe the dynamic response of luminescent centers and distinguish point defect from CsPbBr_3_ nanocrystals are needed. The challenges and controversies in perovskites have also brought us a great opportunity to test new theory, develop new experimental techniques and eventually provide us new understanding and insight to develop and engineer better materials for a wide range of optoelectronic device applications.
